# Characterization of Non-Monotonic Relationships between Tumor Mutational Burden and Clinical Outcomes

**DOI:** 10.1158/2767-9764.CRC-24-0061

**Published:** 2024-07-08

**Authors:** Jordan Anaya, Julia Kung, Alexander S. Baras

**Affiliations:** 1 Department of Pathology, Johns Hopkins University School of Medicine, Baltimore, Maryland.; 2 Biomedical Informatics and Data Science, Johns Hopkins University School of Medicine, Baltimore, Maryland.; 3 The Sidney Kimmel Comprehensive Cancer Center, Johns Hopkins University School of Medicine, Baltimore, Maryland.; 4 Bloomberg∼Kimmel Institute for Cancer Immunotherapy, Sidney Kimmel Comprehensive Cancer Center, Johns Hopkins University School of Medicine, Baltimore, Maryland.

## Abstract

Potential clinical biomarkers are often assessed with Cox regressions or their ability to differentiate two groups of patients based on a single cutoff. However, both of these approaches assume a monotonic relationship between the potential biomarker and survival. Tumor mutational burden (TMB) is currently being studied as a predictive biomarker for immunotherapy, and a single cutoff is often used to divide patients. In this study, we introduce a two-cutoff approach that allows splitting of patients when a non-monotonic relationship is present and explore the use of neural networks to model more complex relationships of TMB to outcome data. Using real-world data, we find that while in most cases the true relationship between TMB and survival appears monotonic, that is not always the case and researchers should be made aware of this possibility.

**Significance:**

When a non-monotonic relationship to survival is present it is not possible to divide patients by a single value of a predictor. Neural networks allow for complex transformations and can be used to correctly split patients when a non-monotonic relationship is present.

## Introduction

When searching for features predictive of survival across different cancer types, researchers often use Cox regression (1–3). The Cox model provides a high level of interpretability in the form of the regression coefficients, but these coefficients simply describe the linear relationship of the feature to the predicted log partial hazard (4). Often there is not a clear reason to assume a linear (and more importantly monotonic) relationship; furthermore, neural nets have previously been proposed as a more flexible approach in this context (5), with some recent applications appearing in the literature (6–9).

Tumor mutational burden (TMB) is a commonly investigated biomarker in the context of immunotherapy (10–16), and its prognostic value has also been investigated in the context of heterogeneous treatments (17, 18). When investigating TMB as a biomarker, researchers often bin patients into a “TMB low” group and a “TMB high” group, which implicitly assumes a monotonic relationship of TMB with survival, independent of the number of bins used. In this case, the relationship is assumed to be a step function with patients below a certain threshold having a certain risk and patients above the threshold having another. The monotonic assumption is that change in risk only increases (or only decreases) with the value of the predictor variable in question. However, other types of relationships could easily be envisioned. For example, while an increase in mutations may initially increase the pathogenicity of a tumor, too many mutations may be detrimental for tumor growth. Alternatively, high or low values of TMB could be a proxy for a different population of samples, such as high TMB being associated with microsatellite instability.

Given enough parameters and data, neural networks are able to learn arbitrarily complex functions. Similar to how we previously leveraged this flexibility to more optimally model the calibration of next-generation sequencing gene panel-derived estimates of exomic TMB (19), we wondered whether such flexible modeling approaches could be applied to characterizing the relationship of TMB with clinical outcomes data. While a single-cutoff approach may work well for monotonic relationships, it would be expected to be suboptimal in the case of a truly non-monotonic relationship. In such a scenario, the lowest and highest TMB values would have similar risk, and the moderate values would be associated with a different risk. In this study, we explored different approaches to attempt to better characterize these more complex relationships and investigated whether such relationships exist in the context of TMB and clinical outcomes data, both in the prognostic sense and predictive sense.

## Materials and Methods

Code for reproducing the results in this manuscript is available at https://github.com/OmnesRes/tmb_survival and has been archived at Zenodo: https://doi.org/10.5281/zenodo.10966684.

### Modeling

Cox-PH models were implemented with lifelines version 0.27.7 (20) and the neural nets with TensorFlow 2.12 (arXiv 1603.04467). TMB values were log transformed before inclusion into the models.

Looking at the formula for hazard in the Cox proportional hazards model, it is easy to see that it is a function of a linear combination of the predictors (4):

$$\lambda \;\left(t|\text{X}\right)\;=\;\lambda_{0}\left(t\right)\;\exp\left(\beta \;\text{X}\right)$$

The hazard for a given set of predictors **X** (X_0_, X_1_, ...) at time *t* is given by a baseline hazard λ_0_(*t*) and a partial hazard exp (β **X**). The log partial hazard is then β **X** (β_0_X_0_ + β_1_X_1_...). In our case, the only predictor is TMB so the log partial hazard is given by β_TMB_ TMB. In order for the partial hazard to be a more complex function of TMB, the variable would have to undergo a transformation prior to inclusion into a Cox model, but the transformation that should be performed is unknown. For an example of a simple polynomial transformation, see Supplementary Fig. S1.

For the neural nets, we interpret the output to be the log partial hazard and use it to calculate the negative partial likelihood, as previously proposed (5). The neural net is essentially a universal function approximator and it transforms the input variable (TMB) into a risk score for each sample. The neural nets were designed to have two dense layers of 128 with softplus activation and a dropout of 0.05, followed by a dense to 1 with no activation. A batch normalization layer was utilized to keep the output values centered at 0.

When training with the real-world datasets, the TMB values in the top 1% were discarded to avoid training with extreme values. Stratified K-fold training was performed with 10 train/test splits and stratified by whether the TMB value was in the top 20th percentile to ensure a somewhat consistent range of TMB values across training splits. For each fold the ranks of the test fold data were recorded in order to calculate the concordance indexes of the models. To ensure a comparable loss calculation between the neural net and Cox models, lifelines was used to calculate all losses, with the predicted risks from the neural nets being passed into a Cox model from lifelines.

For the cutoff analyses, we found the optimal cutoffs as determined by logrank statistic by exhaustively searching every possible data split. For the single-cutoff analyses, we required each group to have at least 25 samples in it, and for the double cutoff analyses, we required the moderate group to have at least 50 samples and the low and high groups to each have at least 25 samples.

### Data processing

Simulated survival data were generated by utilizing an exponential distribution (21), and a uniform distribution was used for censoring with approximately 30% of the data censored. For the simulated data, only TMB values below 64 were used as it was difficult to prevent extreme simulated risks for our quadratic relationship, which highlights a potential issue of using polynomials for modeling data with a long tail.

When generating the log partial hazards for the simulated data, TMB values were first log transformed. For the linear simulated data, the risks (log partial hazards) were set to 0.5 × TMB. For the non-monotonic data, we set the risks to [exp[−(2 TMB−1.5)^2^/(4 TMB)]−0.6] × 2. For the quadratic data, we set the risks to [(−[TMB−2]^2^) × 15 + 40] × 0.05. For the step data, if the TMB value was below the median TMB value then the risk was set to 0, otherwise it was set to 1.

The Cancer Genome Atlas (TCGA) somatic mutation calls were processed as previously described to calculate exomic TMB (19). We used all available panels in the Biopharma Collaborative (BPC) data except for VICC-01-SOLIDTUMOR and UHN-48-V1 due to their small sizes. We used GENIE release 14.1 (22) to obtain somatic mutations and panel coordinates and defined TMB as nonsynonymous mutations per Mb of panel coding sequence. PyRanges (23) was used to perform intersections. For BPC, we only included patients whose pathology procedure occurred within 180 days of diagnosis.

### Data availability

Publicly available data generated by others were used by the authors. TCGA somatic calls (24) were obtained from https://gdc.cancer.gov/about-data/publications/mc3-2017. TCGA clinical data were obtained from Liu and colleagues (25). MSK datasets were obtained from Samstein and colleagues and Valero and colleagues (15, 18). BPC data are available at https://www.synapse.org/#!Synapse:syn27056700. GENIE 14.1 panel information was obtained from https://www.synapse.org/#!Synapse:syn52918985. The GFF3 is available at ftp://ftp.ensembl.org/pub/grch37/current/gff3/homosapiens/Homosapiens.GRCh37.87.gff3.gz. The Broad coverage WIGs are available at https://www.synapse.org/#!Synapse:syn21785741.

## Results

**Figure 1:**
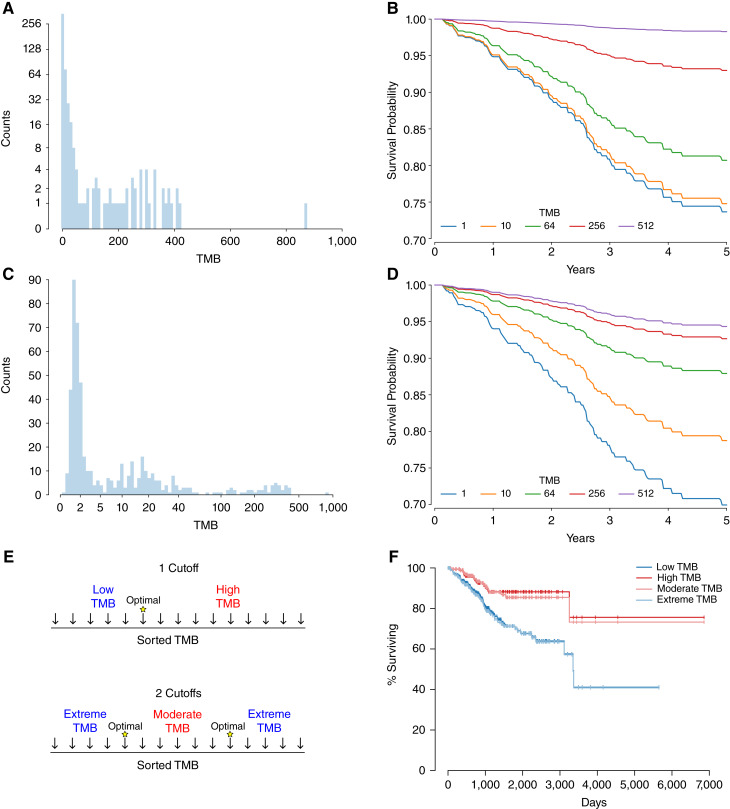
TMB transformation strategies. **A,** the TMB distribution for TCGA UCEC data. **B,** predicted survival curves for several TMB values for a univariate Cox model. **C,** distribution for a log transformation of the TMB values. **D,** the resulting survival curves for a univariate Cox model with the log-transformed values. **E,** two strategies for creating a binary label based on TMB, a single-cutoff approach and a two-cutoff approach. **F,** the resulting survival curves for the most optimal cutoffs with both binary label approaches.

Given the long right-tailed distribution of TMB (Fig. 1A), when modeling the relationship of this data to the log partial hazard, some form of a transformation will generally be required as these extreme values multiplied by the estimated model coefficient will generate unrealistic partial hazards. We can demonstrate this by fitting a Cox model to untransformed TMB values for uterine corpus endometrial carcinoma (UCEC) and the corresponding survival data in TCGA dataset (25). Generating predicted survival curves for several different TMB values (Fig. 1B), we see that what the field would consider large differences in TMB result in minimal differences in the curves, and at the highest TMB values, patients are predicted to survive longer than humanly possible.

One solution to an extreme variable distribution is to log transform the data (Fig. 1C). We see that fitting a Cox model to this transformed data now generates distinct survival curves for TMB values 1 and 10 (Fig. 1D), but the survival curves for the largest values are still unrealistic and this method does not provide a recommendation for how to split patients.

A common transformation in the field is to create a binary label, and the TMB value that determines this label is viewed as the optimal cutoff (Fig. 1E). However, both the Cox regression approach and the optimal cutoff approach make the assumption the risk only increases or decreases with respect to TMB. We introduce a two-cutoff approach that defines a “moderate” group and then combines the low and high groups into one “extreme” group. Depending on the data and the cutoffs selected, the different cutoff approaches can give very similar answers (Fig. 1F).

### Simulated data reveals limitations of current approaches

**Figure 2:**
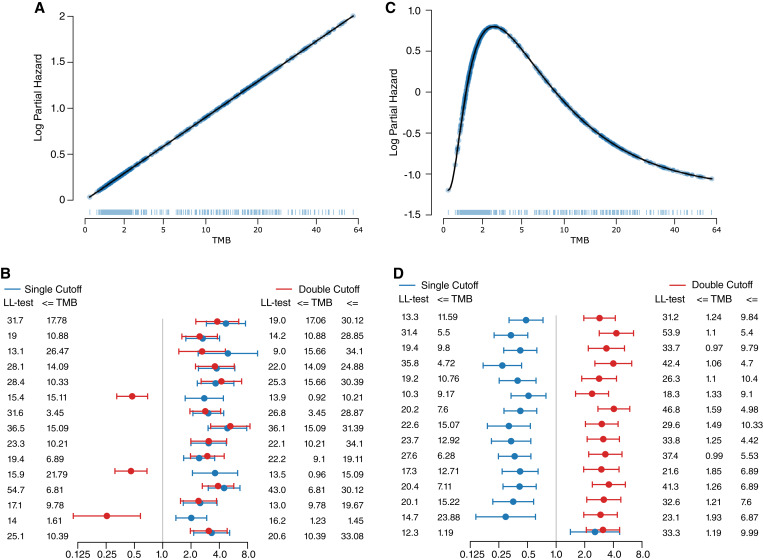
Cutoff analysis with simulated data. Fifteen simulated survival datasets were generated for either a monotonic relationship with TMB (**A**) or a non-monotonic relationship (**C**). **B,** shows the hazard ratios and associated log-likelihood ratio tests and associated cutoffs of searching for an optimal cutoff or an optimal two cutoffs for a monotonic relationship, while **(D)** shows the results with non-monotonic data. In (**A** and **C**), the TMB distribution is shown as a rug plot with the true risks shown as a scatterplot. In (**B** and **D**), each row represents a different simulated dataset.

In Fig. 1, we were using actual TCGA survival data so we did not know whether the single-cutoff or two-cutoff approach was the correct way to model the data. To determine the validity of our new two-cutoff approach, we can turn to simulated data. In this simulated data, the TMB values are real to mimic the distribution and sample sizes we would see in actual data (the values of the UCEC TCGA samples), but the survival data are generated according to a known function mapping logged TMB values to log partial hazard. When applying a single-cutoff approach to data with a linear relationship (Fig. 2A), the approach correctly identified a “TMB high” group with a significant hazard ratio for every simulation (Fig. 2B).

Although a single-cutoff approach works well for this data, we wondered what would happen with a more complex relationship (Fig. 2C). Further, we wanted to understand if our two-cutoff approach could be used to identify this type of relationship. While two cutoffs have been proposed before (26), in those contexts three groups were generated where the “TMB mid” group was expected to have a risk in between that of “TMB low” and “TMB high,” which retains the monotonic nature of the relationship. In contrast, in our two-cutoff approach, we assign the same risk to the “TMB low” and “TMB high” groups with the “TMB mid” displaying a different survival risk, which represents a non-monotonic relationship that can be described as a “Goldilocks effect” wherein we have some cases where the TMB is too “cold,” some cases where it is too “hot,” and in between where it is “just right”.

In the case of a monotonic relationship, we would expect grouping the highest and lowest values together would result in poor correlation, and when we applied the new two-cutoff approach to the monotonic data, the statistical significance was often lower than the single-cutoff approach (Fig. 2B), with the “TMB mid” group often having the higher hazard.

However, in the case of a non-monotonic relationship, grouping the lowest and highest values is exactly what should be done and when these two different approaches were applied to data simulating a non-monotonic relationship of TMB with survival, we found the two-cutoff approach correctly associated a middle group with a larger hazard and always did so with greater statistical significance than the single-cutoff approach (Fig. 2D).

These results give a potential framework for identifying non-monotonic relationships of an input variable to survival—if the single-cutoff approach has a better test statistic, then the relationship is possibly monotonic, and if the two-cutoff approach has a better test statistic, the relationship is possibly non-monotonic. However, while we generally saw this expected pattern in the simulated data, it was not always the case, and this heuristic does not reveal the true underlying relationship, such as a linear relationship versus a step function or others. Instead of comparing different transformation strategies (one cutoff vs. two), we can simply allow a fully connected neural network (FCN) to learn the relationship between predictor variable and outcome measures directly from the available data.

We looked at the first two simulated survival datasets for both the linear and non-monotonic relationships and compared the results of a FCN to a Cox regression implemented with lifelines. To compare the model fits, we looked at the C-index and log-likelihood (a larger value is better in both cases) for data in the test folds of 10 stratified K-folds and also compared these metrics to what would be obtained with the ground truth relationship (Table 1). With regards to the linear data, both the Cox model and the FCN had metrics nearly indistinguishable from the ground truth, but for the non-monotonic data, the FCN had noticeably better metrics than the Cox model.

**Table 1:** Simulated data metrics

		Linear data		Non-monotonic data	
Simulated dataset	Model	LL score	C-index	LL score	C-index
1 (Row 1)	Ground Truth	−0.895	0.601	−0.905	0.651
	Cox-PH	−0.895	0.601	−0.929	0.528
	FCN	−0.896	0.601	−0.909	0.646
2 (Row 2)	Ground Truth	−0.921	0.620	−0.872	0.665
	Cox-PH	−0.921	0.620	−0.892	0.580
	FCN	−0.922	0.620	−0.884	0.645

Log-likelihood and C-indexes for the test folds of two representative monotonic and non-monotonic simulated datasets from Fig. 2 for either a Cox model or a neural network.

**Figure 3:**
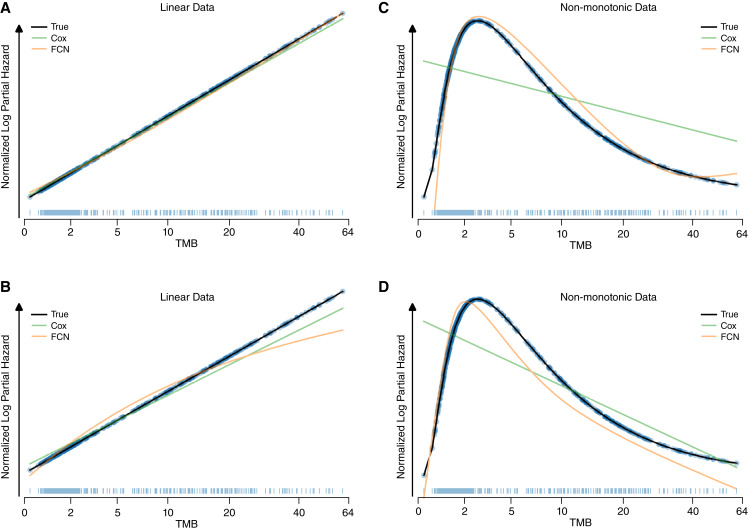
Simulated data model fits. Mean-normalized model fits from a Cox model and a neural net were averaged over 10 K-folds for the first two simulated survival datasets for a linear and non-monotonic relationship. **A** and **B,** monotonic data. **C** and **D,** non-monotonic data. TMB distributions shown as rug plots with the true risks shown as a scatterplot.

We also visualized the model fits of the FCNs, Cox regressions, and the true underlying relationships (Fig. 3). Despite having a large number of parameters, our FCN produced a very similar fit to a Cox regression for the linear data, with both closely following the true relationship (Fig. 3A and B). Visually, the extra parameters of the FCN allowed it to correctly follow the shape of the non-monotonic data (Fig. 3C and D), while the Cox model was a poor fit to the data, which is consistent with the model metrics. For additional data distributions, see Supplementary Fig. S1. For the variability in model fits across training folds, see Supplementary Fig. S2.

### Applying neural networks to TCGA

The simulated data gave us confidence that if the relationship strongly deviates from a monotonic relationship, then an FCN will be able to better model this and this would be reflected in the C-index and model log-likelihood metrics. Our next question was whether this tool would allow us to find evidence for such “Goldilocks effects” in real-world datasets.

Using the mutation call and survival data for solid tumors in TCGA dataset, we compared the model metrics of a Cox model and our FCN (Table 2).

**Table 2:** TCGA data metrics

	Cox-PH		FCN	
Cancer	LL score	C-index	LL score	C-index
BLCA	−1.337	0.573	−1.338	0.570
CESC	−0.593	0.512	−0.600	0.490
ESCA	−0.842	0.556	−0.842	0.556
GBM	−1.967	0.491	−1.967	0.478
HNSC	−1.381	0.532	−1.379	0.540
KIRC	−0.643	0.616	−0.643	0.616
KIRP	−0.372	0.573	−0.372	0.573
LGG	−0.630	0.712	−0.625	0.712
LIHC	−0.953	0.572	−0.955	0.572
LUAD/LUSC	−1.488	0.514	−1.488	0.514
OV	−1.415	0.569	−1.416	0.569
PAAD	−1.177	0.551	−1.193	0.553
COAD/READ	−0.670	0.580	−0.670	0.580
SKCM	−1.346	0.578	−1.322	0.617
STAD	−1.203	0.559	−1.204	0.559
UCEC	−0.515	0.551	−0.521	0.562

Log-likelihood scores and C-indexes for the test folds of different cancers in TCGA for a Cox model and a neural net.

**Figure 4:**
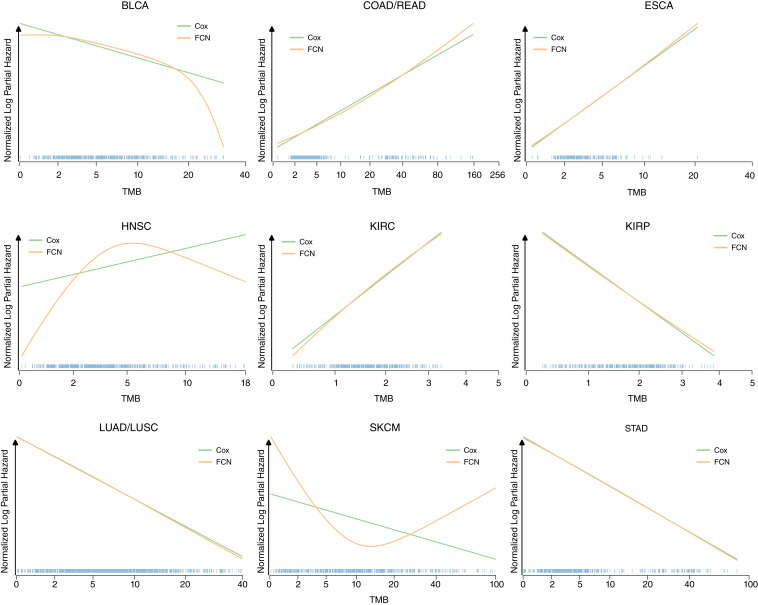
TCGA model fits. Nine selected cancer types are shown. Mean-normalized Cox and neural net model fits were averaged over 10 K-folds. TMB distributions shown as rug plots.

In most cancer types, there was limited difference between a Cox model and an FCN, suggesting the relationship likely is monotonic (or in some cases simply no relationship). However, in skin cutaneous melanoma (SKCM), the FCN displayed a noticeably better log likelihood and C-index. SKCM is a cancer type where immunotherapy is recommended and where TMB has been suggested as predictive biomarker (27, 28). Looking at some of the model fits, we see tumor types where the FCN predicted a perfectly linear relationship and followed the Cox predictions, cases where the FCN has slight deviations from the Cox predictions, and then SKCM where the FCN predicts a strong concave up relationship (Fig. 4).

### Applying neural networks to immunotherapy datasets

TCGA dataset contains patients receiving the standard of care at the time and includes patients at different stages of the disease. In this heterogeneous context, exploring the relationship of TMB with survival is more closely associated with some form of prognostication rather than predicting response to a given therapeutic modality. In contrast, when investigating a cohort in which a specific treatment has been administered we generally are considering a biomarker that is predictive of response. It is in this context that TMB has often been explored as a biomarker and many approaches effectively attempt to stratify the cohort into “TMB low” and “TMB high” and compare clinical outcome measures (response, disease-free survival, etc.). Given our findings of a non-monotonic relationship of survival for SKCM in TCGA dataset, we wondered if we would identify non-monotonic relationships in other datasets, either in a prognostic or predictive context.

The AACR Project GENIE BPC recently released clinical data for patients with lung and colon cancer. Using the corresponding panel mutational data for these patients, we applied our model to a lung cohort treated without immunotherapy (BPC NSCLC NonIO), a lung cohort treated with immunotherapy (BPC NSCLC IO), and a colon cohort treated without immunotherapy (BPC colorectal NonIO). Similar to TCGA data, our neural network did not detect non-monotonic relationships in these cancer types (Table 3; Supplementary Fig. S3).

**Table 3:** BPC and MSK data metrics

	Cox-PH		FCN	
Data	LL score	C-index	LL score	C-index
BPC NSCLC NonIO	−1.183	0.568	−1.183	0.552
BPC NSCLC IO	−1.486	0.532	−1.486	0.531
BPC Colorectal NonIO	−1.645	0.511	−1.643	0.477
MSK SKCM IO (2019 cohort)	−1.026	0.565	−1.028	0.566
MSK NSCLC IO (2019 cohort)	−1.883	0.527	−1.871	0.576
MSK NSCLC IO (2021 cohort)	−2.043	0.514	−2.037	0.551
MSK NSCLC NonIO (2021 cohort)	−1.297	0.610	−1.294	0.610
MSK Colorectal NonIO (2021 cohort)	−1.001	0.484	−0.995	0.565
MSK Pancreatic NonIO (2021 cohort)	−2.044	0.577	−2.044	0.577
MSK Endometrial NonIO (2021 cohort)	−0.375	0.648	−0.379	0.649

Log-likelihood scores and C-indexes for the test folds of different cancers in the BPC and MSK data for a Cox model and a neural net.

Memorial Sloan Kettering (MSK) has released several large datasets of patients assayed with their gene panel and the corresponding clinical information. Without some form of common identifier across these datasets it is difficult to know how much patient overlap exists between the data releases, but we applied our FCN to a 2019 dataset that only included patients treated with immunotherapy (15) and a 2021 dataset that had both IO naive and IO treated patients (18). While we had observed TMB to have a non-monotonic relationship with survival in TCGA dataset for SKCM, in the IO treated MSK melanoma cohort, we did not observe a difference between a Cox regression and our FCN (Table 3). This is consistent with a monotonic relationship of TMB and clinical outcome in the context of melanoma treated with IO and supports a TMB-high versus TMB-low stratification in this context. The FCN model showed better performance over Cox modeling in the non-IO treated colorectal cancer cohort (MSK Colorectal NonIO), in which a concave down model was apparent (Supplementary Fig. S4). Some improvement in performance was seen in the MSK non–small cell lung cancer cohorts treated with IO, and the observed relationship was also concave down (Supplementary Fig. S4).

### Example of model application

The benefit of directly modeling the relationship with survival is that any deviations from a linear fit will be accounted for in the model predictions. This allows a researcher wanting to identify a cutoff for splitting patients to simply use a cutoff based on model risks. Then, working backward from the model risks the corresponding input variable cutoff(s) can be found. If the relationship of the input variable to survival is non-monotonic, then a single model risk will result in two input variable values, while a monotonic relationship will result in a single input variable cutoff.

**Figure 5:**
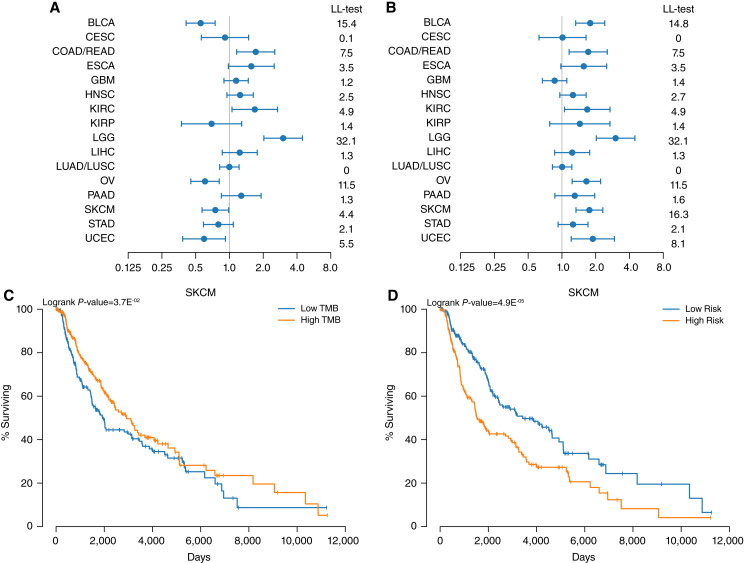
Neural network can create a better binary label. Patients were split by either median TMB (**A** and **C**) or median risk from a neural net (**B** and **D**). **A** and **B** show the hazard ratios and log-likelihood tests of each cancer type for these splits while **C** and **D,** show the Kaplan curves for SKCM with these splits along with the logrank *P*-value.

We can demonstrate what this might look like by splitting each cancer in TCGA dataset by either the median TMB value or median FCN model risk value. When splitting by median TMB, the higher TMB group may or may not have a higher hazard, while when splitting by median model risk, the higher risk group should have a higher hazard. Ignoring this difference in sign, in most cases there is almost no difference in the test statistic as the FCN predicted a monotonic relationship for most cancers (Fig. 5A and B). However, for SKCM, we see that a single cutoff of TMB is inappropriate. In fact, with a median cutoff, the relationship of TMB to survival is barely significant while it is highly significant with a FCN model output-based risk cutoff (Fig. 5C and D).

## Discussion

Identifying consistent cutoffs for biomarkers has always been challenging (29) as only relationships that resemble a step function result in stable optimal cutoff values (30), a result recapitulated in our Fig. 2; Supplementary Fig. S1. It is possible to identify a step function with a neural net (Supplementary Fig. S1; Supplementary Table S1), but here we used neural nets to investigate yet another consideration when identifying potential biomarkers and cutoffs: the assumption of a monotonic relationship may not hold. If this scenario occurs, it has several important implications. First, when no relationship is found with a Cox regression, a very strong relationship could still be present. Second, the predictions for values at the extremes, i.e., patients who would be predicted to have the best or worst survival according to a Cox model, will contain the largest errors (see the fits for SKCM in Fig. 4). Third, if a non-monotonic relationship is present, then a single cutoff is inappropriate and two-cutoffs of the input variable will be needed to split patients into the appropriate two groups.

When modeling data one can make strong assumptions about the underlying relationship (often simple models with few parameters) or the data can be allowed to dictate the relationship (often complex models with a high number of parameters). This is known as the bias/variance tradeoff. A standard Cox regression makes a strong assumption about the relationship between input and risk, i.e., it is linear, whereas a neural net learns the relationship from the data, which may be nearly linear or more complex. The potential downfall of an approach such as a neural net is that it may detect relationships in the training data that are specific to those samples and not representative of the true underlying relationship. In the modeling presented in this paper, we had a single input (TMB) and a single output (risk), with relatively shallow networks with dropout. As can be seen in our simulated experiments, with the sample sizes used we did not observe any significant overfitting to the training data. However, if an input was multidimensional, the network may need to be carefully constructed or more samples may be needed.

The possibility of a non-monotonic relationship is not just theoretical. While approaches to correlate TMB to survival have generally assumed a monotonic relationship, using a more flexible approach we have identified multiple datasets where the relationship between TMB and survival appears more complex. This study has a few important limitations, the first of which is that we did not undertake a more complex multivariate modeling of clinical outcome using features such as age, stage, grade, etc. This was done on purpose as the intent of this work is to highlight how the relationship of TMB to clinical outcome can be modeled to account for potential non-monotonic relationships and not to comprehensively approach clinical outcome modeling across the relevant predictors. The second important limitation is related in that we did not design these experiments such that we would be “validating” a specific TMB↔clinical outcome correlation across tumor type and clinical setting. Again, this was intended given the modeling focus of the work. We believe what we have developed and presented herein has important implications for studies that investigate TMB as a predictive and/or prognostic biomarker. Further, we show that fairly simple neural networks like we have presented here can help avoid the pitfalls of ordinary Cox-PH based regression with respect to the potential of non-monotonic relationships.

## Supplementary Material

Figure S1 Simulated step data and examples with a polynomial transformation. 15 simulated survival datasets were generated for a step relationship with TMB (A). B shows the hazard ratios and associated log-likelihood ratio tests and associated cutoffs of searching for an optimal cutoff, while C shows the fits of a Cox model, FCN neural network, and a neural network comprised of a single neuron with sigmoid activation. D shows an example of a quadratic relationship with TMB (E). Generating a simulated dataset from the risk relationship in D we explored fitting a Cox model with a two degree polynomial in addition to a neural net. In F we show the fit of a two degree polynomial with non-monotonic data.

Figure S2 Model variability across folds. A, mean-normalized model fits for each training fold for an FCN model with simulated linear data. B, mean-normalized model fits for each training fold for an FCN model with simulated non-monotonic data.

Figure S3 BPC fits. Cox and neural net model fits were mean normalized and averaged over 10 K-folds. TMB distributions shown as rug plots.

Figure S4 MSK fits. Cox and neural net model fits were mean normalized and averaged over 10 K-folds. TMB distributions shown as rug plots.

Table S1 Simulated step function metrics. Log-likelihood and C-indexes for the test folds of a simulated step function dataset for either a Cox model, a FCN neural network, and a neural network comprised of a single neuron with sigmoid activation.
