# Supplementary figures and images for: Characterization of Non-Monotonic Relationships between Tumor Mutational Burden and Clinical Outcomes
Source: Cancer Res Commun. 2024 Jul 8;4(7):1667–76. doi: 10.1158/2767-9764.CRC-24-0061 (PMC11229404; doi:10.1158/2767-9764.CRC-24-0061)

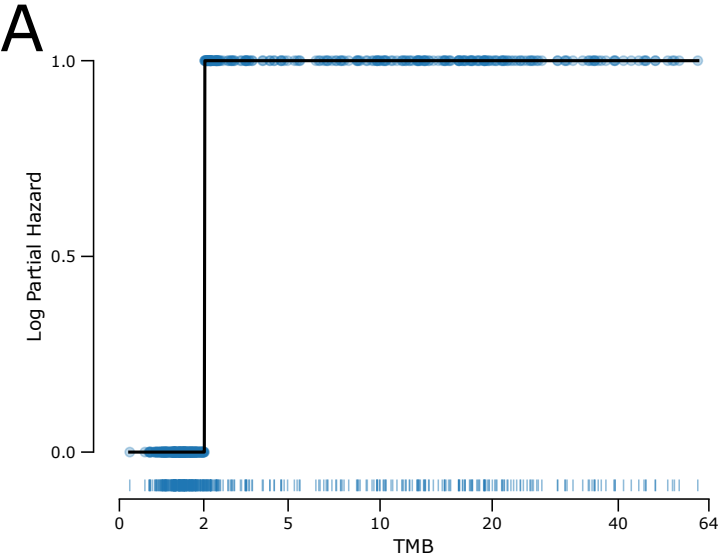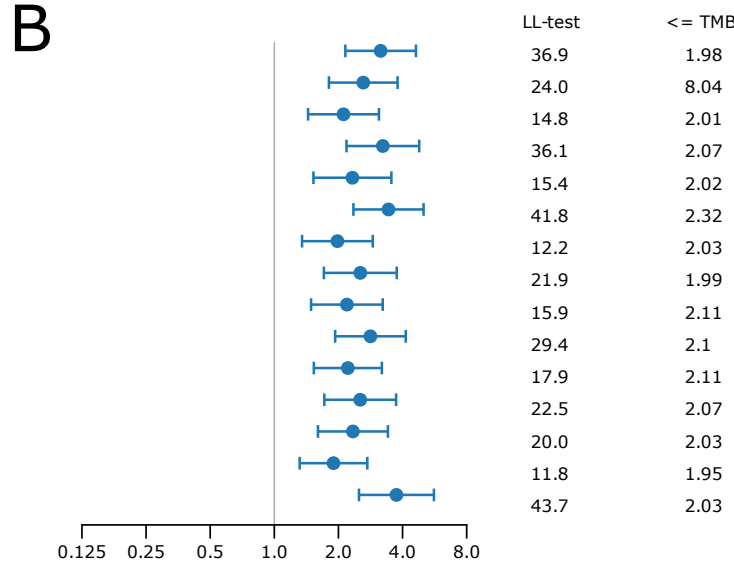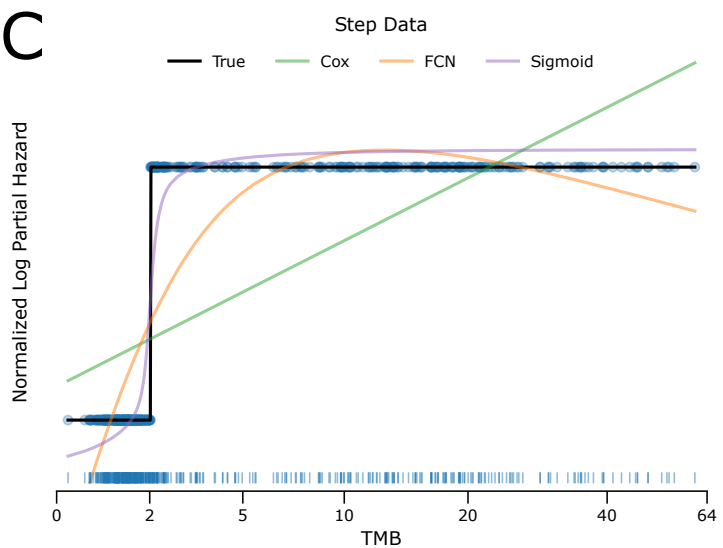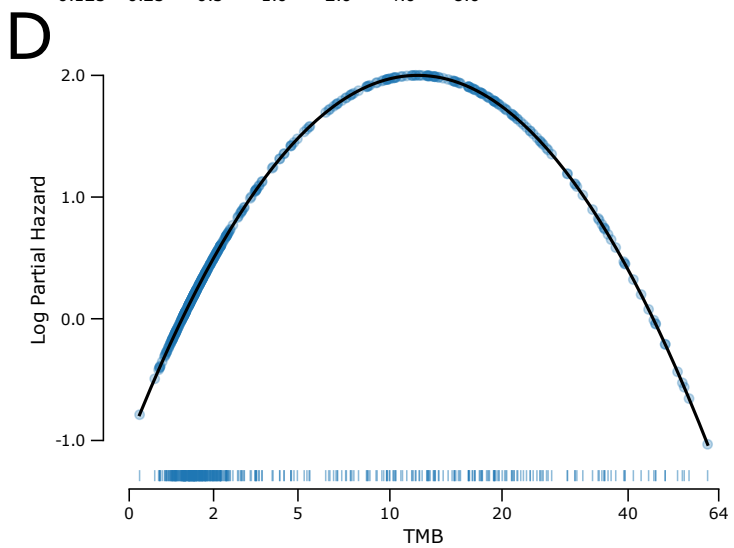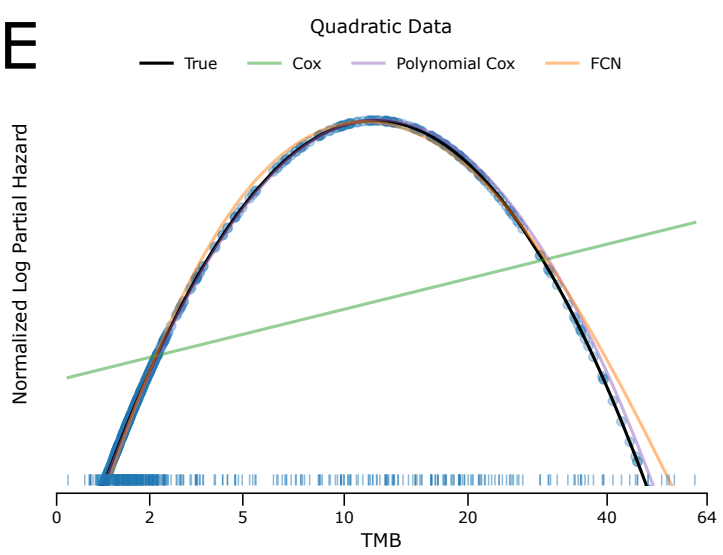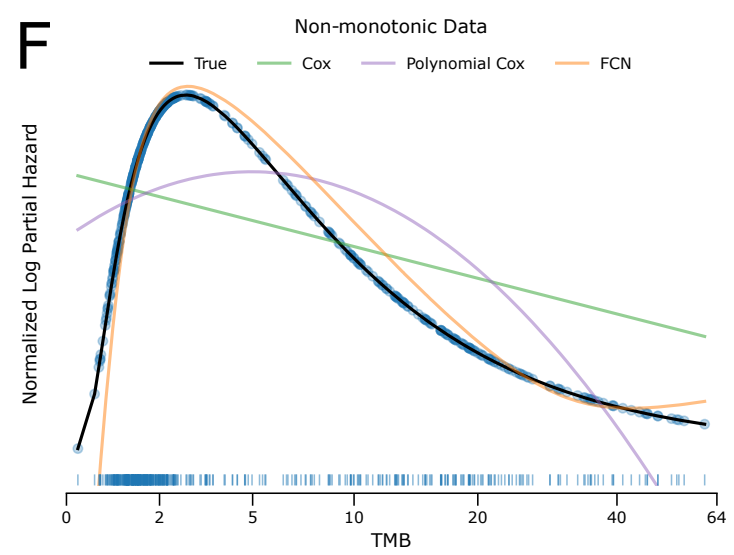

Supplementary Figure 1. Simulated step data and examples with a polynomial transformation.

Supplement: Figure S1 — Simulated step data and examples with a polynomial transformation. 15 simulated survival datasets were generated for a step relationship with TMB (A). B shows the hazard ratios and associated log-likelihood ratio tests and associated cutoffs of searching for an optimal cutoff, while C shows the fits of a Cox model, FCN neural network, and a neural network comprised of a single neuron with sigmoid activation. D shows an example of a quadratic relationship with TMB (E). Generating a simulated dataset from the risk relationship in D we explored fitting a Cox model with a two degree polynomial in addition to a neural net. In F we show the fit of a two degree polynomial with non-monotonic data. [file crc-24-0061_figure_s1_suppsf1.pdf]

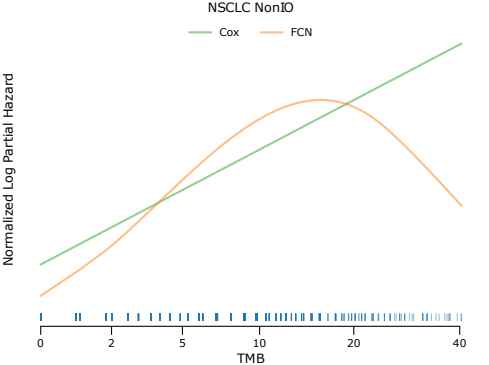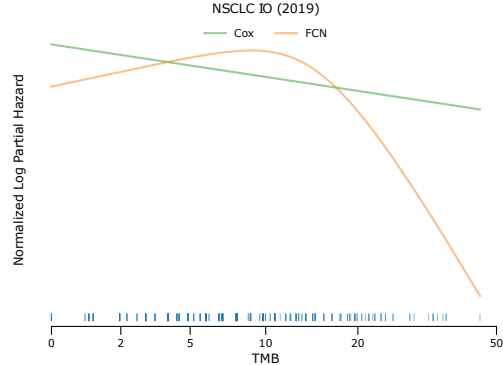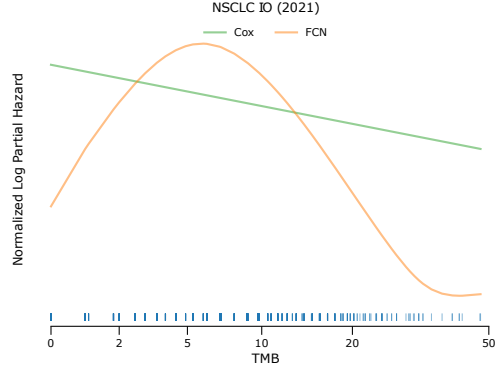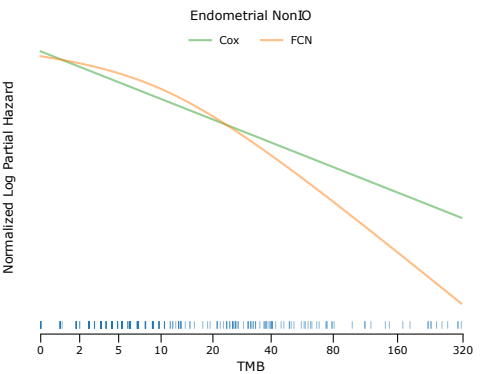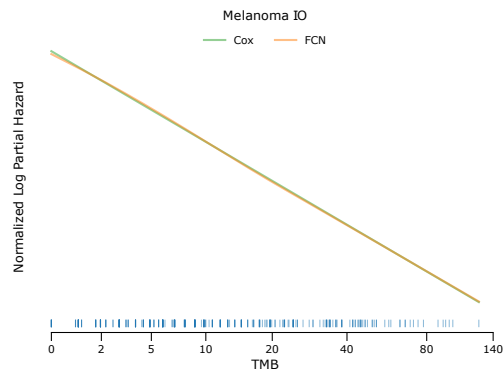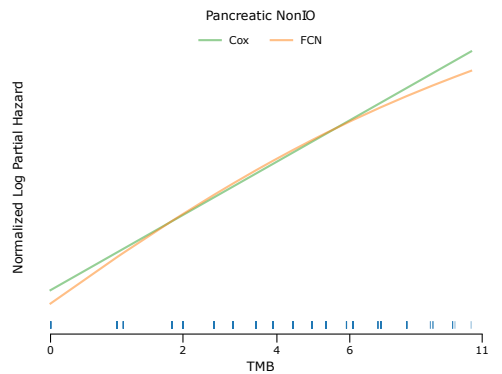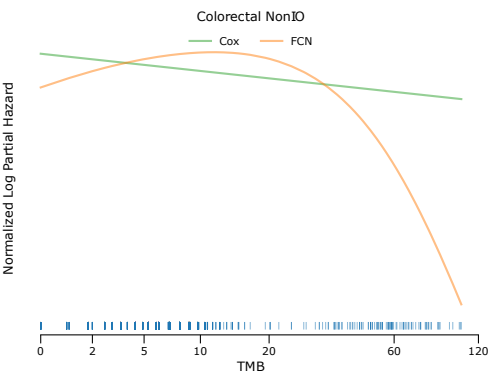

Figure S4. MSK fits. Cox and neural net model fits were mean normalized and averaged over 10 K-folds.

Supplement: Figure S4 — MSK fits. Cox and neural net model fits were mean normalized and averaged over 10 K-folds. TMB distributions shown as rug plots. [file crc-24-0061_figure_s4_suppsf4.pdf]
