# Supplementary material for: Characterization of Non-Monotonic Relationships between Tumor Mutational Burden and Clinical Outcomes
Source: Cancer Res Commun. 2024 Jul 8;4(7):1667–76. doi: 10.1158/2767-9764.CRC-24-0061 (PMC11229404; doi:10.1158/2767-9764.CRC-24-0061)
Supplement: Figure S2 — Model variability across folds. A, mean-normalized model fits for each training fold for an FCN model with simulated linear data. B, mean-normalized model fits for each training fold for an FCN model with simulated non-monotonic data. [file crc-24-0061_figure_s2_suppsf2.pdf]

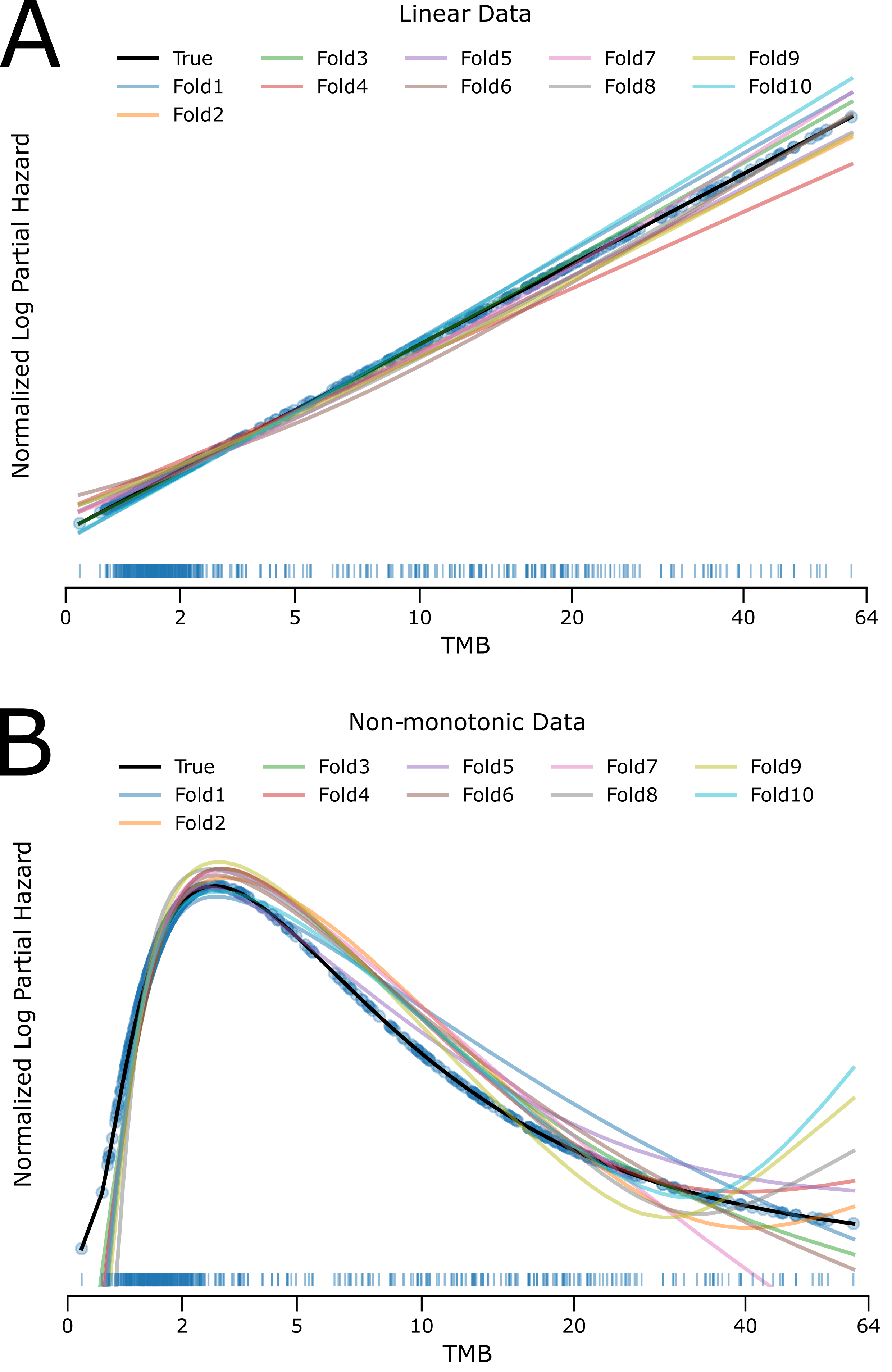
A
Linear Data
True
Fold1
Fold2
Fold3
Fold4
Fold5
Fold6
Fold7
Fold8
Fold9
Fold10
Normalized Log Partial Hazard
0
2
5
10
20
40
64
TMB
B
Non-monotonic Data
True
Fold1
Fold2
Fold3
Fold4
Fold5
Fold6
Fold7
Fold8
Fold9
Fold10
Normalized Log Partial Hazard
0
2
5
10
20
40
64
TMB

Supplementary Figure 2. Model variability across folds.
