# Supplementary material for: Characterization of Non-Monotonic Relationships between Tumor Mutational Burden and Clinical Outcomes
Source: Cancer Res Commun. 2024 Jul 8;4(7):1667–76. doi: 10.1158/2767-9764.CRC-24-0061 (PMC11229404; doi:10.1158/2767-9764.CRC-24-0061)
Supplement: Figure S3 — BPC fits. Cox and neural net model fits were mean normalized and averaged over 10 K-folds. TMB distributions shown as rug plots. [file crc-24-0061_figure_s3_suppsf3.pdf]

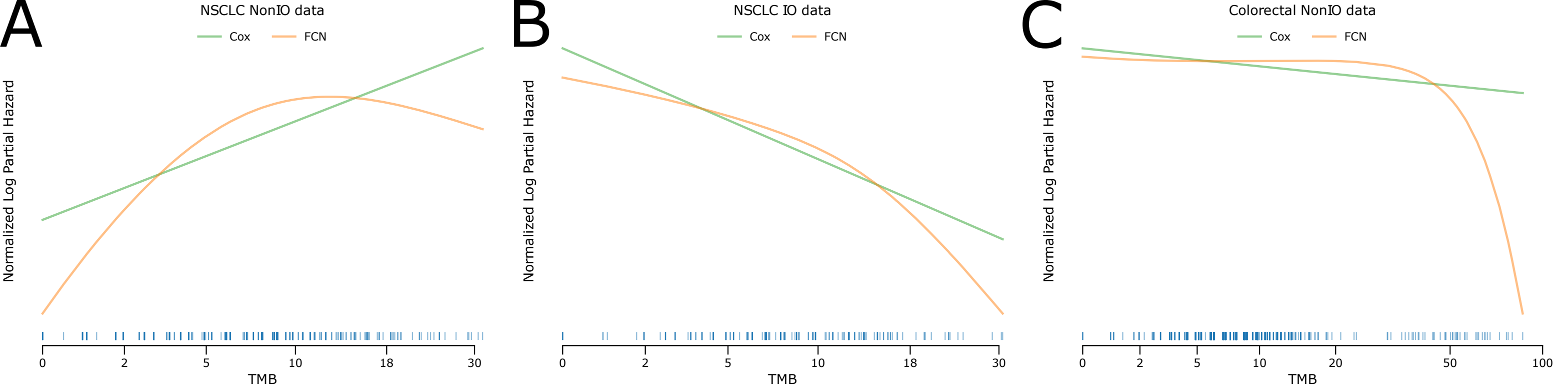
A
NSCLC NonIO data
Cox
FCN
Normalized Log Partial Hazard
0
2
5
10
18
30
TMB
B
NSCLC IO data
Cox
FCN
Normalized Log Partial Hazard
0
2
5
10
18
30
TMB
C
Colorectal NonIO data
Cox
FCN
Normalized Log Partial Hazard
0
2
5
10
20
50
100
TMB

Figure S3. BPC fits. Cox and neural net model fits were mean normalized and averaged over 10 K-folds.
